# Extended-Spectrum Beta-Lactamase- and Carbapenemase-Producing Enterobacteriaceae Family of Bacteria from Diarrheal Stool Samples in Northwest Ethiopia

**DOI:** 10.1155/2022/7905350

**Published:** 2022-03-08

**Authors:** Minichil Worku, Michael Getie, Feleke Moges, Alem Getaneh Mehari

**Affiliations:** ^1^University of Gondar Comprehensive Specialized Hospital Laboratory, Gondar, Ethiopia; ^2^University of Gondar, College of Medicine and Health Sciences, School of Biomedical and Laboratory Sciences, Department of Medical Microbiology, Gondar, Ethiopia

## Abstract

**Background:**

Resistance among the commensal flora is a serious threat because they are highly populated ecosystems like the gut, maybe a source of extraintestinal infections. Infections due to extended-spectrum beta-lactamase (ESBL)- and carbapenemase (CPM)-producing Enterobacteriaceae family of bacteria impose a major global issue because they are usually resistant to multiple antimicrobial agents. Data on the fecal ESBL- and CPM-producing group of bacteria in developing countries including Ethiopia are limited mainly due to resource constraints. Thus, this study aimed to determine the prevalence of multidrug-resistant (MDR)-, ESBL-, and CPM-producing Enterobacteriaceae family of bacteria from diarrheal stool samples at the University Hospital, Northwest Ethiopia.

**Materials and Methods:**

A hospital-based cross-sectional study was conducted involving a total of 384 study participants having gastrointestinal complaints from January to April 2019. A diarrheal stool sample was aseptically collected and inoculated on a MacConkey agar plate. After getting pure colonies, biochemical and antimicrobial susceptibility testing was done following standard microbiological techniques. ESBL production was screened using ceftazidime and cefotaxime and confirmed using a combined disk diffusion test. Carbapenemases were screened by meropenem disk and confirmed by the modified carbapenem inactivation method. Data were checked, cleaned, and entered using Epi Info version 7.1 and transferred to SPSS version 20 for analysis.

**Result:**

A total of 404 Enterobacteriaceae groups of bacteria were isolated from 384 diarrheal stool samples. The overall prevalence of fecal MDR-, ESBL-, and CPM-producing group of Enterobacteriaceae was 196 (48.5%), 66 (16.3%), and 4 (1%), respectively. Of the total ESBL-producing Enterobacteriaceae, *E. coli* (41/66 (62.1%)) and *K. pneumoniae* (18/66 (27.3%)) were the most predominant isolates. One half of CPE has been observed in *Citrobacter* species and the rest in *E. coli* (25%) and *P. vulgaris* (25%). *Conclusion and Recommendation*. Finding the high rate of ESBL-producing Enterobacteriaceae and CPE requires strict infection control measures and careful selection of empirical therapy in the study area. Therefore, active surveillance with large sample size and better infection prevention control is needed.

## 1. Introduction

Enterobacteriaceae are a group of Gram-negative, rod-shaped facultative anaerobe, and their natural host is the human and animal intestine [1, 2]. The human gastrointestinal tract is a reservoir for pathogens causing infections including urinary tract infections, nosocomial infections, skin, and soft tissue infections. Bacterial translocation is the invasion of indigenous intestinal bacteria through the gut mucosa to normal sterile tissues and the internal organs. Resistance among the commensal flora is a serious threat because they are highly populated ecosystems like the gut, maybe a source of extraintestinal infections, and resistant strains may spread to other hosts or transfer genetic resistance elements to other members of microorganisms [3, 4]. Colonization of the gastrointestinal tract plays a key role in the epidemiology and clinical significance of extended-spectrum beta-lactamase (ESBL)- and carbapenemase (CPM)-producing bacteria [5].

Extended-spectrum beta-lactamase-producing Enterobacteriaceae have been reported worldwide since the early 1980s [6]. In the past decade, there has been an alarming increase in antibiotic-resistant Enterobacteriaceae producing ESBL due to the overuse of broad-spectrum cephalosporins [7]. Fecal ESBL-producing Enterobacteriaceae in the community was first reported in Spain and Poland in 2001 and 2002, respectively [8].

Extended-spectrum beta-lactamase-producing Enterobacteriaceae have worldwide distributions with varying degrees of prevalence in the community and hospitals [9, 10]. Infections due to ESBL-PE and CPM-producing Enterobacteriaceae represent a major global health threat because they are usually resistant to multiple antimicrobial agents and lack carbapenem drugs [11, 12]. Although antimicrobial resistance is a global problem, the impact is higher in sub-Saharan Africa due to limited available resources for healthcare infrastructure and wide irrational use of antimicrobial agents. From those who take antibiotics, more than one-third do not get prescriptions from a doctor and about a quarter obtain antibiotics from an informal dispenser [13, 14].

Currently, infections due to ESBL- and CPM-producing Enterobacteriaceae are concerning for many reasons including increased hospital costs, length of stay, treatment failure, and mortality rates [15]. ESBL- and CPM-producing Enterobacteriaceae are difficult to treat because of high levels of resistance to many antibiotics that break down all *β*-lactam agents including carbapenems and make them ineffective [16]. The prevalence of ESBL- and CPM-producing Enterobacteriaceae is on the rise worldwide [17–19]. Screening Enterobacteriaceae for ESBL and CPM production is essential for better antibiotic selection and preventing its further emergence and spread [9]. However, data on the fecal ESBL- and CPM-producing Enterobacteriaceae are limited in developing countries, especially in Ethiopia, particularly Gondar due to resource constraints. So, this research intended to determine the magnitude of the intestinal ESBL- and CPM-producing Enterobacteriaceae family of bacteria at the University of Gondar Comprehensive Specialized Hospital.

## 2. Materials and Methods

### 2.1. Study Design, Area, and Period

A hospital-based cross-sectional study was conducted at the University of Gondar Comprehensive Specialized Hospital in Gondar Town, Northwest Ethiopia, from January to April 2019. The town is located 737 km far from Addis Ababa, the capital city of Ethiopia, and 180 km far from Bahir Dar, the regional capital. According to the central and statistical agency of Ethiopia report in 2015, the town has twelve sub-city, twenty-two urban, and eleven rural kebeles with a total projected population of 323,900. There are 8 health centers, 21 private clinics, and one primary hospital in the town. The hospital provides healthcare services for more than 5 million people living in North, South, and West Gondar Zones, as well as urban and rural kebeles surrounding the town. The hospital has 518 beds and from 350 to 400 patients visit the hospital each day and from 100 to 120 emergency patients visit the hospital each day. The hospital also has four emergency suites with a triage unit for distribution.

### 2.2. Study Population

All gastrointestinal tract complaint patients who visited the University of Gondar Comprehensive Specialized Hospital during the study period were the study population.

### 2.3. Sample Size and Sampling Technique

The sample size was determined using the single population proportion formula:

(1) $$\begin{array}{c} n=\frac{{\left(Z\alpha /2\right)}^{2}*p*\left(1-p\right)}{d^{2}}, \end{array}$$

where *n* = sample size. The prevalence is taken from the previous study which is from Addis Ababa, Ethiopia p = 0.52. At 95% CI Zα/2 = 1.96.

By taking the prevalence of ESBLs and carbapenem resistance, Enterobacteriaceae infection was conducted at Tikur Anbessa Specialized Hospital, which showed 0.52 [13].

(2) $$\begin{array}{c} n=\frac{{\left(1.96\right)}^{2}*\left(0.52\right)*\left(1-0.52\right)}{{\left(0.05\right)}^{2}}=383.54∼384. \end{array}$$

Based on this proportion, a total of 384 diarrheal stool samples from patients with gastrointestinal complaints were collected using a convenient sampling technique.

## 3. Data Collection and Laboratory Methods

### 3.1. Sociodemographic Characteristics

After taking written informed consent and verbal assent from the study participants, sociodemographic data were collected using a predesigned questionnaire.

#### 3.1.1. Specimen Collection and Processing

The study participants were instructed to collect approximately 2 grams of diarrheal stool into a clean, leak-proof container. The specimen of the study participants was collected at the University of Gondar Comprehensive Specialized Hospital laboratory. Each stool sample was immediately transported to the School of Bio-Medical and Laboratory Sciences, Biomedical Complex of Medical Microbiology Section, using Cary Blair transport media, and following aseptic technique, a loop full of the diarrheal sample was inoculated onto a MacConkey agar (Oxoid, *Code*: CM0115) and incubated aerobically at 37°C for 16–24 hours.

### 3.2. Identification

#### 3.2.1. Preliminary Identification

The preliminary identification of bacteria was based on the colony characteristics of the organisms and Gram staining characteristics. The Gram stain is a very important preliminary step in the initial identification of bacteria based on staining characteristics and morphology.

#### 3.2.2. Biochemical Tests

The biological tests were performed on isolated colonies for the identification of Enterobacteriaceae based on their biochemical reaction. These biochemical tests include triple sugar iron agar to test microorganism's ability to ferment sugars (glucose, lactose, and sucrose), to produce hydrogen sulfide (H2S) and gas production, indole test to detect the ability of an organism to produce indole from tryptophan present in the medium, citrate utilization test to detect whether the organism can use sodium citrate as the sole source of carbon for metabolism and growth, urease production test to detect organisms that produce urease enzyme, lysine decarboxylase test to determine the enzymatic ability of an organism to decarboxylate or hydrolyze an amino acid to form an amine with the liberation of carbon dioxide, and motility test to differentiate species of bacteria that are motile and interpret the result based on their biochemical reaction [20].

#### 3.2.3. Drug Susceptibility Testing

Modified Kirby Bauer disk diffusion technique using a Muller Hinton agar (MHA) (Oxoid, UK) was used for antimicrobial susceptibility tests. Bacterial suspension of three to five isolated colonies was done using 0.85% normal saline, and the turbidity was adjusted at 0.5% McFarland standard. Using a sterile cotton applicator stick, the suspension had been inoculated on MHA and left at room temperature for 3–5 minutes until it becomes dry. Then, different antibiotic disks including ceftazidime (30 *μ*g) and cefotaxime (30 *μ*g) were applied on inoculated MHA and incubated for 24 hr at 37°C. Ceftazidime (30 *μ*g) and cefotaxime (30 *μ*g) disks were used for presumptive identification of ESBL production. The zones of inhibition were measured by a ruler, and the results were interpreted as susceptible, intermediate, and resistant using CLSI 2019 and 2020 performance standards for antimicrobial susceptibility testing interpretation table. The zone of inhibition ≤22 mm for ceftazidime and ≤27 mm for cefotaxime was considered as potential ESBL producers [21, 22].

### 3.3. Laboratory Test for the Detection of ESBL and CPM

#### 3.3.1. Confirmatory Test for ESBL Producer

The potential ESBL-producing Enterobacteriaceae was confirmed by the combined disk method. Colony suspension of suspected ESBL-producing Enterobacteriaceae was inoculated onto MHA, and then, ceftazidime (30 *μ*g) and ceftazidime-clavulanic acid (30/10 *μ*g), cefotaxime (30 *μ*g), and cefotaxime-clavulanic acid (30/10 *μ*g) disks were placed at 20 mm distance apart. If *a* ≥5 mm increase in zone diameter for either antimicrobial agent was tested in combination with clavulanate vs the zone diameter of the agent when tested alone, it was confirmed as ESBL-producing Enterobacteriaceae [21, 22].

#### 3.3.2. Screening Test for CPM

Carbapenemase-producing Enterobacteriaceae was screened using Meropenem disks. Colony suspension of isolated bacteria was inoculated onto MHA, and then, Meropenem (10 *μ*g) disks were placed and incubated at 37°C for 24 hrs. If the zone of inhibition is ≤19 mm, it was considered as a potential CPM-producing Enterobacteriaceae [21].

#### 3.3.3. Confirmatory Test for CPM

The suspected CPM is confirmed by the modified carbapenem inactivation method (mCIM). The isolated bacterial colony, which was suspected for CPM, was diluted with 2 ml of Trypticase soy broth, and meropenem (10 *μ*g) disk was immersed in the suspension and then incubated for 4 hours. A standard strain of meropenem susceptible *E. coli* ATCC 25922 was suspended in 0.85% normal saline, compared with McFarland standard (1 : 10 dilution), and then the whole plate of MHA is inoculated. After 4 hrs of incubation, the meropenem disk was removed from the test tube and placed on the MHA plate, which was inoculated by *E. coli* ATCC 25922 meropenem-sensitive strain and incubated at 37°C for 18–24 hours. After incubation, if the zone of inhibition diameter is between 6–15 mm and 16–18 mm with a pinpoint colony, it was considered as carbapenem resistance Enterobacteriaceae [21, 22].

### 3.4. Operational Definitions

*ESBL producers* are bacteria that can produce the enzymes, which confer resistance to most beta-lactam antibiotics [1].

*MDR* is defined as resistance to three or more different classes of antibiotics [13].

*Carbapenemases* are beta-lactamase enzymes that inactivate almost all hydrolyzable beta-lactam antibiotics including the carbapenems [1].

*Gastrointestinal tract complain* is discomfort in the gastrointestinal tract with abdominal cramp, diarrhea, vomiting, and distension of the abdomen [23].

### 3.5. Laboratory Quality Control

All media were prepared according to the manufacturer's instruction and following standard operational procedures. All materials, equipment, and procedures were adequately controlled based on preanalytical, analytical, and postanalytical stages of quality assurance that were incorporated in standard operating procedures at the School of Bio-Medical and Laboratory Sciences of Bio-Medical Complex of Medical Microbiology Section. Culture media were checked for sterility by incubating 5% batch of the media at 37°C for 24 hours, and the performance test was checked by inoculating known control strains of *Escherichia coli* ATCC 25922 and *K. pneumoniae ATCC® 700603* to confirm consistency of materials, methods, and results. *K. pneumoniae* ATCC^*BAA*^1705 and ATCC^*BAA*^ 1706 were used as positive and negative quality control, respectively, for carbapenemase production.

### 3.6. Data Analysis and Interpretation

Data were collected, coded, and entered into EPI Info version 7 to check completeness and clearance and then transferred to SPSS version 20 for analysis. The relevant findings of the study were described using text and summarized using frequencies and percentages. Data were also presented using tables and figures.

## 4. Results

### 4.1. Sociodemographic Characteristics of the Study Participants

Of 384 study participants, 200 (52.1%) were males. The mean age of the study subjects was 30.76 ± SD: 16.93. The highest frequency age group of the study participants was 16–30 years (170 (44.1%)), and most (225 (58.6%)) of the study participants were urban residents. The majority (310 (80.7%)) of the study participants were from the outpatient department, while the remaining were from inpatients (Table 1).

### 4.2. Prevalence of Enterobacteriaceae

A total of 404 Enterobacteriaceae group of bacteria were isolated from 384 diarrheal stool samples. *E. coli* was the predominate (219 (54%)) isolate followed by *K. pneumoniae* (50 (12%)), *K. ozaenae* (15 (3.7%)), *Citrobacter species* (14 (3.5%)), *Shigella* species (10 (2.5%)), *E. cloacae* (7 (1.7%)), *Proteus* species (6 (1.5%)), *Serratia species* (6 (1.5%)), *E. aerogenes* (1 (0.2%)), *S. typhi* (1 (0.2%)), and other Enterobacteriaceae groups (75 (18.6%)) (Table 2).

### 4.3. Antimicrobial Susceptibility Patterns of Enterobacteriaceae

From the total AST performed, the highest resistance was observed in tetracycline (254 (64%)), ampicillin (172 (53%)), cotrimoxazole (166 (41%)), and amoxicillin/clavulanic acid (152 (40%)), whereas the highest sensitivity has occurred in cefepime (330 (82%)), gentamicin (313 (79.8%)), tobramycin (306 (78%)), and ciprofloxacin (299 (74%)). Of the total isolates of *E. coli*, 155/219 (71%) were resistant to tetracycline followed by ampicillin (149 (68%)), but these isolates were highly sensitive to gentamicin (184 (84%)), cefepime (177 (81%)), cefixime (177 (81%)), tobramycin (175 (80%)), cefoxitin (174 (79%)), and ciprofloxacin (165 (75%)). *K. pneumoniae* was resistant to tetracycline (41/50 (82%)), ampicillin/clavulanic acid (36 (72%)), cotrimoxazole (31 (62%)), and ceftazidime (28 (56%)), but sensitive to gentamicin (37 (74%)), cefoxitin (30 (60%)), ciprofloxacin (35 (70%)), tobramycin (30 (60%)), and cefuroxime (33 (66%)) (Table 2).

### 4.4. Drug-Resistant Patterns of Enterobacteriaceae

From the total of 404 isolated Enterobacteriaceae group of bacteria, 196 (48.5%) (95% CI: 43.3–53.5) were multidrug-resistant (MDR). Among these, *E. coli* accounts the highest (118 (60.2%)), followed by *K. pneumoniae* (37 (18.9%)), *K. ozaenae* (12 (6.1%)), *Citrobacter* species (10 (5.7%)), *Proteus* species (6 (3.1%)), *Serratia* species (5 (2.5%)), and *E. cloacae* (4 (2.1%)) (Table 3).

### 4.5. Prevalence of ESBL- and Carbapenemase-Producing Enterobacteriaceae

**Figure 1:**
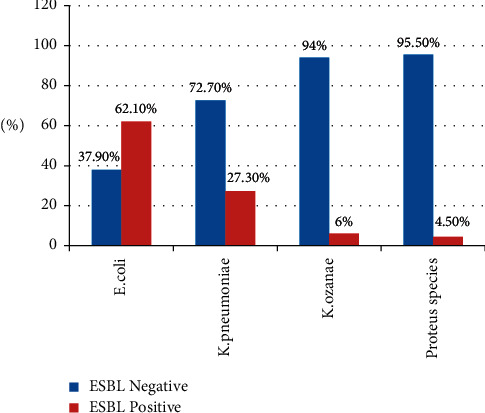
Distribution of ESBL-producing Enterobacteriaceae family of bacteria from diarrheal stool samples at the University of Gondar Comprehensive Specialized Hospital from January–April 2019.

Among the total 404 isolated Enterobacteriaceae group of bacteria, 106 (26.3%) were screened positive and 66 (16.3%) (95% CI: 12.9–20.0) were confirmed positive for ESBL production. Of confirmed (66) ESBL-producing Enterobacteriaceae group of bacteria, *E. coli* account for the highest (41 (62.1%)) followed by *K .pneumoniae* (18 (27.3%)), *K. ozaenae* (4 (6%)), and *Proteus* species (3 (4.5%)). As per CLSI 2019 Guideline, other Enterobacteriaceae groups were excluded from ESBL detection because they have no breakpoint in the guidelines (Figure 1).

A total of 105 Enterobacteriaceae group of bacteria (66 confirmed ESBL-producing Enterobacteriaceae and the rest 39 other Enterobacteriaceae) were screened for CPM production using Meropenem disk. Among these, 4 (3.8%) were presumptive CPM producers. These four presumptive CPM producers were confirmed by the modified carbapenem inactivation method (mCIM), and all (4/4 (100%)) were CPM-producing group of Enterobacteriaceae. From the total CPM-producing group of Enterobacteriaceae, *Citrobacter* species accounted for 2 (50%), *E. coli* accounted for 1 (25%), and *P. vulgaris* accounted for 1 (25%) (Table 4). In total, 4/404 (0.9%) CPM producers were detected.

## 5. Discussion

The gut microbiota is a reservoir of antimicrobial resistance genes that are thought to contribute to the emergence of multidrug-resistant pathogens through horizontal gene transfer [24]. The emergence of antimicrobial resistance, the main cause of morbidity and mortality from otherwise treatable infections, is largely attributed to the inappropriate use of antimicrobials [25]. To counteract the spread of antimicrobial resistance, it is paramount to know which organisms harbor mobile antimicrobial resistance genes and which organisms engage in horizontal gene transfer [26]. In this study, we investigated the occurrence of multidrug-resistant (MDR) Enterobacteriaceae group of bacteria and ESBL- and CPM-producing Enterobacteriaceae group of bacteria in human fecal flora from patients with gastrointestinal complaints. From the total of 384 diarrheal stool samples, 404 Enterobacteriaceae family of bacteria were isolated and the overall prevalence of MDR Enterobacteriaceae was 48.5% (95% CI: 43.3%–53.5%), which is comparable with a study done in Norway (48%) [11]. However, our result is lower than a study done in Mozambique University (88%) [27], but it was slightly higher than a study conducted in Addis Ababa (43%) [13], Morocco (42.8%) [28], and India (12.4%). The irrational use of antibiotics is a huge problem in Ethiopia, and many bacteria were resistant to commonly used antibiotics, and similarly, multidrug-resistant bacterial strains are numerous [29].

Infections due to ESBL-producing Gram-negative bacteria have led to increased mortality, morbidity, and economic burden worldwide. These bacteria can colonize the healthy intestine of human beings and can disseminate in communities and hospitals [30]. Dissemination of ESBL-producing Enterobacteriaceae family of bacteria to healthy people has increased dramatically worldwide [31]. Asymptomatic carriage of ESBL-producing pathogens might act as a source of infection in both the community and hospitals [30]. In this study, the overall prevalence of ESBL-producing Enterobacteriaceae group of bacteria was 16.3% (95% CI: 12.9%–20.0%), which was concordant with a report in France (17.7%) [32], Mozambique University (20%) [27], and Norway (15.8%) [11]. However, it was lower than a report in Addis Ababa (52%) [13], Egypt (65%) [12], Morocco (42.8%) [28], Tanzania (34.3%) [33], Beirut (24.5%) [34], Southeast Asia (50.7%) [6], Venezuela (34.6%) [35], Turkey (30%) [36], Sweden (35%) [37], and Korea (28%) [38]. This variation may be due to poor diagnostic facilities in the study area; for example, antibiotic susceptibility testing was not performed by combining with the automated VITEK®2 system or other advanced techniques. The other reasons might be due to the difference in the study population and geographical location. In contrast, the current finding was higher than a study conducted in Amsterdam (8.6%) [10] and Switzerland (5.8%) [39]. This variation may be due to the improper use of antibiotics in the population, geographical location, and poor personal and environmental hygienic practices.

*E. coli* and *K. pneumoniae* are common species of Enterobacteriaceae that both have pathogenic potential and that frequently incorporate ESBL-encoding genes. The high prevalence rate of ESBL-producing *E. coli* and *K. pneumoniae* fecal carriage and high level of multidrug resistance among ESBL-producing *E. coli* and *K. pneumoniae* were demonstrated [40]. In this study, 62.1% (95% CI: 50.0%–72.7%) of *E. coli* was isolated, which was in line with a report in Addis Ababa (70%) [13] and Mozambique University (62%) [27], but lower than a study done in Norway(86%) [11] and Southeast Asia (97%) [6]. However, it was higher than a study conducted in Morocco (48.5%) [28]. This discrepancy of isolation may be due to the differences in geographical location, study populations, sample size, and methodological variability that could bring variation in the prevalence.

Screening for carriage of CPEs in stool in patients undergoing elective or emergency gastrointestinal surgical procedures, in patients with hematological malignancies taking chemotherapy, or patients with planned bone marrow transplantation can guide clinicians about gut colonization of multidrug-resistant Enterobacteriaceae as these groups of patients are at risk of possible endogenous infection [3]. In this study, we also screened and investigated the occurrence of CPM-producing Enterobacteriaceae family of bacteria in human fecal flora from patients with gastrointestinal complaints. This can help in starting appropriate prophylactic antibiotics if required. Clinicians and microbiologists must be aware of the prevalence of CPM-producing isolates in the human intestinal tract as these types of drug-resistant strains are potential sources of endogenous infections. Overall, the percentage of fecal CPM-producing Enterobacteriaceae family of bacteria recovered was 1% (95% CI: 0.2%–2.0%), which is comparable to those reported from other parts of the world like Korea (0.3%) [38]. As a result, this study is lower than a study conducted in Morocco (13%) [28], Uganda 10% [41], India (6.6%) [4], and Mexico (16.6%) [42]. However, this study reported a higher prevalence than a study done in Norway (0%) [11].

Hundred percent resistance to ceftazidime and cefotaxime was observed in *E. coli,* which is compatible with a study conducted in Madagascar that showed 100% resistance to ceftazidime and cefotaxime [43], Addis Ababa ceftazidime (97%) and cefotaxime (98%) [13], and Turkey cefotaxime (96%) and ceftazidime (94%) [36], but it was higher than a study conducted in Venezuela ceftazidime (46%) and cefotaxime (68.7%) [35], and Guinea-Bissau ceftazidime (66%) and cefotaxime (65%) [44]. This variation may be due to the difference in diagnostic technique, indiscriminate use of antibiotics, the number of patients that attended each hospital, disease exposure, geographic differences among the study participants, the type of healthcare activities, and infection control practices in the hospitals. Similarly, *K. pneumoniae* was 100% resistant for both ceftazidime and cefotaxime, respectively, which is in concordant with a study conducted in Guinea-Bissau ceftazidime (97.8%) and cefotaxime (97.8%) [44], and Tanzania ceftazidime (97%) and cefotaxime (98.6%) [33].

Limitation: advanced techniques such as VITEK®2 system were not used for drug susceptibility testing.

## 6. Conclusion and Recommendation

Multidrug resistance-, extended-spectrum beta-lactamase-, and carbapenemase-producing Enterobacteriaceae family of bacteria were higher in gastrointestinal tract infections. *E. coli* followed by *K. pneumoniae* was the most predominant ESBL-producing Enterobacteriaceae family of bacteria. Many isolates showed higher sensitivity to aminoglycosides (gentamicin and tobramycin), cephalosporin (cefoxitin and cefepime), and quinolones (ciprofloxacin). Finding the high rate of ESBL production in Gram-negative bacteria requires strict infection control measures and careful selection of empirical therapy in the study area. Antimicrobial susceptibility tests should also be performed for isolates of Enterobacteriaceae. ESBL- and CPM-producing Enterobacteriaceae family of bacteria should be screened if MDR Enterobacteriaceae is isolated or if the patient is at high risk to improve the infection prevention practice and to minimize cross-transmission in healthcare settings. Active surveillance with a large sample size will be better to know the high prevalence of ESBL and CPM producers in gastrointestinal tract infections.

## Figures and Tables

**Table 1:** Sociodemographic characteristics of patients with gastrointestinal complaints at the University of Gondar Comprehensive Specialized Hospital, Northwest Ethiopia, January–April 2019.

Characteristics		Number (%)
Gender	Male	200 (52.1)
	Female	184 (47.9)

Age category (yearss)	≤5	28 (7.3)
	6–15	32 (8.3)
	16–30	170 (44.3)
	31–45	77 (20.1)
	46–60	52 (13.5)
	>61	25 (6.5)

Residence	Rural	159 (41.4)
	Urban	225 (58.6)

Occupation	Farmer	65 (16.9)
	Civil servant/employee	94 (24.5)
	Private	27 (7.0)
	Housewife	48 (12.5)
	Student	100 (26.0)
	Others	50 (13.0)

Educational level	Illiterate	89 (23.1)
	Primary school	102 (26.6)
	Secondary school	46 (12.0)
	Higher education	119 (31)
	N/A	28 (7.3)

Marital status	Married	192 (50.0)
	Single	184 (47.9)
	Divorced	8 (2.1)

Department	Inpatient	74 (22.9%)
	Outpatient	310 (80.7%)

**Table 2:** Antimicrobial susceptibility patterns of bacterial isolates identified from diarrheal stool samples at the University of Gondar Comprehensive Specialized Hospital from January–April 2019.

Antibiotic disks	*E. coli* = 219	*K. pneumoniae* = 50	*K. ozaenae* = 15	*Citrobacter* = 14	*P. vulgaris* = 6	*E. cloacae* = 7	*S. typhi* = 1	*Shigella* species = 10	*Serratia* = 6	Others = 75	*E. aerogenes* = 1	Total = 404
		No (%)	No (%)	No (%)	No (%)	No (%)	No (%)	No (%)	No (%)	No (%)	No (%)	No (%)	No (%)
AMP	S	44 (20)	—	1 (7)	—	—	—	0 (0)	5 (50)	—	17 (42)	—	67 (23.5)
	I	26 (12)	—	1 (7)	—	—	—	0 (0)	2 (20)	—	22 (55)	—	51 (17.9)
	R	149 (68)	—	13 (86)	—	—	—	1 (100)	3 (30)	—	1 (3)	—	167 (58.6)

AMC	S	71 (32)	8 (16)	5 (33.3)	—	0 (0)	—	0 (0)	6 (60)	—	23 (57)	—	113 (33.1)
	I	49 (22)	6 (12)	5 (33.3)	—	2 (33.3)	—	0 (0)	1 (10)	—	17 (43)	—	80 (23.5)
	R	99 (46)	36 (72)	5 (33.3)	—	4 (66.7)	—	1 (100)	3 (30)	—	0 (0)	—	148 (43.4

FEP	S	177 (81)	24 (48)	13 (86.7)	13 (93)	5 (83.3)	6 (86)	1 (100)	10 (100)	6 (100)	39 (97)	1 (100)	295 (79.9)
	I	24 (11)	13 (26)	2 (13.3)	1 (7)	0 (0)	1 (14)	0 (0)	0 (0)	0 (0)	1 (3)	0 (0)	42 (11.4)
	R	18 (8)	13 (26)	0 (0)	0 (0)	1 (16.7)	0 (0)	0 (0)	0 (0)	0 (0)	0 (0)	0 (0)	32 (8.7)

CTX	S	154 (70)	18 (36)	12 (80)	13 (93)	4 (66.7)	6 (86)	—	—	5 (83.3)	38 (95)	1 (100)	251 (70.1)
	I	11 (5)	5 (10)	0 (0)	1 (7)	0 (0)	0 (0)	—	—	0 (0)	2 (5)	0 (0)	19 (5.3)
	R	54 (25)	27 (54)	3 (20)	0 (0)	2 (33.3)	1 (14)	—	—	1 (16.7)	0 (0)	0 (0)	88 (24.6)

FOX	S	174 (79.4)	24 (48)	14 (93)	—	5 (83.3)	—	—	—	—	40 (97)	—	257 (77.6)
	I	28 (11)	13 (26)	0 (0)	—	1 (16.4)	—	—	—	—	1 (3)	—	43 913.0)
	R	17 (7.6)	13 (26)	1 (7)	—	0 (0)	—	—	—	—	0 (0)	—	31 (9.4)

CXM	S	155 (71)	33 (66)	8 (53.3)	—	—	—	—	—	—	27 (67)	—	223 (68.8)
	I	35 (16)	9 (18)	6 (40)	—	—	—	—	—	—	13 (33)	—	63 (19.4)
	R	29 (13)	8 (16)	1 (6.7)	—	—	—	—	—	—	0 (0)	—	38 (11.7)

CAZ	S	156 (71)	22 (44)	11 (73.3)	14 (100)	5 (83.3)	7 (100)	—	—	4 (66.7)	36 (90)	1 (100)	256 (71.5)
	I	6 (3)	0 (0)	2 (13.3)	0 (0)	0 (0)	0 (0)	—	—	1 (16.6)	4 (10)	0 (0)	13 (3.6)
	R	57 (26)	28 (56)	2 (13.3)	0 (0)	1 (16.7)	0 (0)	—	—	1 (16.6)	0 (0)	0 (0)	89 (24.9)

GEN	S	184 (84)	37 (74)	10 (66.7)	12 (85.7)	5 (83.3)	6 (85.7)	—	—	4 (66.7)	29 (72)	1 (100)	288 (80.4)
	I	17 (7)	8 (16)	5 (33.3)	2 (14.3)	0 (0)	1 (14.3)	—	—	1 (16.6)	11 (28)	0 (0)	45 912.6)
	R	18 (9)	5 (10)	0 (0)	0 (0)	1 (16.7)	0 (0)	—	—	1 (16.6)	0 (0)	0 (0)	25 (7.0)

TOB	S	175 (80)	30 (60)	11 (73.3)	13 (93)	4 (66.7)	6 (71.4)	—	—	5 (83.3)	30 (75)	1 (100)	275 (76.8)
	I	18 (8)	10 (20)	3 (20)	1 (7)	0 (0)	0 (0)	—	—	0 (0)	10 (25)	0 (0)	42 (11.7)
	R	26 (12)	10 (20)	1 (6.4)	0 (0)	2 (33.3)	1 (28.6)	—	—	1 (16.7)	0 (0)	0 (0)	41 (11.5

TET	S	41 (19)	9 (18)	1 (7)	2 (14)	—	1 (14.2)	0 (0)	5 (50)	0 (0)	16 (40)	0 (0)	75 (20.7)
	I	23 (10)	0 (0)	0 (0)	1 (7)	—	2 (28.6)	0 (0)	2 (20)	0 (0)	18 (45)	0 (0)	46 (12.7)
	R	155 (71)	41 (82)	14 (93)	11 (79)	—	4 (57.1)	1 (100)	3 (30)	6 (100)	6 (15)	1 (100)	242 (66.7)

CPR	S	165 (75)	35 (70)	9 (60)	9 (64)	3 (50)	3 (60)	1 (100)	10 (100)	4 (66.7)	28 (75)	1 (100)	268 (73.0)
	I	22 (10)	9 (18)	5 (33.3)	3 (21)	1 (16.7)	1 (20)	0 (0)	0 (0)	2 (33.3)	12 (25)	0 (0)	55 (15.0)
	R	32 (15)	6 (12)	1 (6.7)	2 (15)	2 (33.3)	1 (20)	0 (0)	0 (0)	0 (0)	0 (0)	0 (0)	44 912.0)

SXT	S	100 (46)	13 (26)	2 (13.3)	5 (36)	2 (33.3)	4 (57)	0 (0)	6 (60)	22 (63)	23 (57)	1 (100)	178 (44.7)
	I	23 (10)	6 (12)	1 (6.7)	1 (7)	0 (0)	1 (14.3)	0 (0)	2 (20)	12 (34)	13 (32)	0 (0)	59 (14.8)
	R	96 (44)	31 (62)	12 (80)	8 (57)	4 (66.7)	2 (28.7)	1 (100)	2 (20)	1 (3)	4 (11)	0 (0)	161 (40.5)

AMP: ampicillin, TET: tetracycline, GEN: gentamicin, CMX: cefuroxime, TOB: tobramycin, CPR: ciprofloxacin, AMC: amoxicillin/clavulanic acid, FEP: cefepime, CAZ: ceftazidime, CTX: cefotaxime, FOX: cefoxitin, AZM: azithromycin, and SXT: trimethoprim-sulfamethoxazole. *Note*. Intrinsically resistance antibiotics that are not recommended for isolates were denoted by (—).

**Table 3:** Prevalence of MDR Enterobacteriaceae family of bacteria from diarrheal stool samples at the University of Gondar Comprehensive Specialized Hospital from January to April 2019.

Isolates	Degree of resistance			
	R3	R4	R5	R6
*E. coli* (*N* = 118)	30 (25.4%)	34 (28.8%)	29 (24.5%)	25 (21.2%)
*K. pneumoniae* (*N* = 37)	12 (32.4%)	11 (29.2%)	9 (24.3%)	5 (13.5%)
*K. ozaenae* (*N* = 12)	6 (50%)	3 (25%)	2 (16.6%)	1 (8.3%)
*Citrobacter* species (*N* = 10)	2 (20%)	7 (70%)	1 (10%)	—
*Proteus Vulgaris* (*N* = 6)	4 (66.6%)	1 (16.7%)	1 (16.6%)	—
*E. cloacae* (*N* = 4)	—	2 (50%)	1 (25%)	1 (25%)
*Salmonella* species (*N* = 1)	—	1 (100%)	—	—
*Shigella* species (*N* = 3)	2 (66.7%)	1 (33.3%)	—	—
*Serratia* species (*N* = 5)	3 (60%)	1 (20%)	1 (20%)	—
Total (*N* = 196)	59 (30.1%)	60 (30.6%)	44 (22.4%)	32 (16.3%)

*Note*. R3-6: resistance to 3, 4, 5, and 6 classes of antibiotics, respectively; ≥R3: resistance to 3 or more classes of antibiotics.

**Table 4:** Distribution of ESBL- and CPM-producing Enterobacteriaceae family of bacteria from diarrheal stool samples at the University of Gondar Comprehensive Specialized Hospital from January to April 2019.

Bacterial isolates	ESBL screening *N* (%)		Confirmed ESBL *N* (%)		CPM screening *N* (%)		Confirmed CPM *N* (%)	
	Positive	Negative	Positive	Negative	Positive	Negative	Positive	Negative
*E. coli*	69 (65)	150	41 (62)	28 (70)	1 (25)	40 (40)	1 (25)	0
*K. pneumoniae*	29 (27)	21	18 (27)	11 (28)	0	18 (18)	0	0
*K. ozaenae*	4 (4)	11	4 (6)	0	0	4 (4)	0	0
*Citrobacter*	N/T	N/T	N/T	N/T	2 (50)	12 (12)	2 (50)	0
*Proteus vulgaris*	4 (4)	2	3 (5)	1 (3)	1 (25)	2 (2)	1 (25)	0
*E. cloacae*	N/T	N/T	N/T	N/T	0	7 (7)	0	0
*S. typhi*	N/T	N/T	N/T	N/T	0	1 (1)	—	—
*Shigella* spp.	N/T	N/T	N/T	N/T	0	10 (10)	—	—
*Serratia* spp.	N/T	N/T	N/T	N/T	0	6 (6)	—	—
*E. aerogenes*	N/T	N/T	N/T	N/T	0	1 (1)	—	—
Total	106 (36.5)	184 (63.4)	**66 (62.2)**	40 (37.7)	4 (3.8)	101 (96.2)	**4** (100)	0

N/T = not tested. The bold values indicate the number of confirmed ESBL and CPM-producing Enterobacteriaceae. Confirmed ESBL Producing enterobactericeae = 66 (62.2%). Confirmed CPM-producing enterobactericeae = 4 (100%)

## Data Availability

The datasets used and/or analyzed during this study are available from the corresponding author.

## Abbreviations

ATCC: American Type Culture Collection
CLSI: Clinical and Laboratory Standard Institute
CPM: Carbapenemase
ESBL: Extended-spectrum beta-lactamase
mCIM: Modified carbapenem inactivation method
MDR: Multidrug-resistant.
